# A Novel Extreme Learning Machine Classification Model for e-Nose Application Based on the Multiple Kernel Approach

**DOI:** 10.3390/s17061434

**Published:** 2017-06-19

**Authors:** Yulin Jian, Daoyu Huang, Jia Yan, Kun Lu, Ying Huang, Tailai Wen, Tanyue Zeng, Shijie Zhong, Qilong Xie

**Affiliations:** 1 College of Electronic and Information Engineering, Southwest University, Chongqing 400715, China; jane19960620@email.swu.edu.cn (Y.J.); huiyuancream@email.swu.edu.cn (D.H.); hanhanblala@email.swu.edu.cn (Y.H.); wtl980059723@email.swu.edu.cn (T.W.); zty172623471@email.swu.edu.cn (T.Z.); z1014850605@email.swu.edu.cn (S.Z.); xql1997@email.swu.edu.cn (Q.X.); 2 Chongqing Key Laboratory of Nonlinear Circuits and Intelligent Information Processing, Chongqing 400715, China; 3 High Tech Department, China International Engineering Consulting Corporation, Beijing 100048, China; lvk@ciecc.com.cn

**Keywords:** electronic nose, gas classification, extreme learning machine, multiple kernel learning, weighted kernels, parameter optimization

## Abstract

A novel classification model, named the quantum-behaved particle swarm optimization (QPSO)-based weighted multiple kernel extreme learning machine (QWMK-ELM), is proposed in this paper. Experimental validation is carried out with two different electronic nose (e-nose) datasets. Being different from the existing multiple kernel extreme learning machine (MK-ELM) algorithms, the combination coefficients of base kernels are regarded as external parameters of single-hidden layer feedforward neural networks (SLFNs). The combination coefficients of base kernels, the model parameters of each base kernel, and the regularization parameter are optimized by QPSO simultaneously before implementing the kernel extreme learning machine (KELM) with the composite kernel function. Four types of common single kernel functions (Gaussian kernel, polynomial kernel, sigmoid kernel, and wavelet kernel) are utilized to constitute different composite kernel functions. Moreover, the method is also compared with other existing classification methods: extreme learning machine (ELM), kernel extreme learning machine (KELM), k-nearest neighbors (KNN), support vector machine (SVM), multi-layer perceptron (MLP), radical basis function neural network (RBFNN), and probabilistic neural network (PNN). The results have demonstrated that the proposed QWMK-ELM outperforms the aforementioned methods, not only in precision, but also in efficiency for gas classification.

## 1. Introduction

An electronic nose (e-nose) is a machine devoted to reproducing the smell processing procedure of the mammalian olfactory system, which has played an immensely crucial role in a wide range of realms, such as disease diagnosis [1], food industry [2], agriculture [3], environmental monitoring and protection [4], etc. It incorporates chemical sensing systems (e.g., sensor arrays) and pattern recognition systems (e.g., artificial neural networks). The chemical sensing systems convert information from gases into electrical signals like olfactory receptors would. The pattern recognition systems discriminate the different gases like a central processor would [5]. A general pattern discrimination system may be described as four different phases with specific platforms, namely, the data acquisition phase, the feature extraction phase, the classification phase, and the decision-making strategy phase. The aim of the classifiers is to assign the data to different categories by utilizing the corresponding feature vectors derived from the feature extraction system [6]. Typically, according to specific mathematical rules, the classifiers are first trained using the data of known volatiles that are related to a set of classes held in a knowledge base. Then, the data of an unknown volatile are tested in the knowledge base and the predicted class is provided. The issues of how to effectively capture the “fingerprint” of the smell and how to classify the chemical volatile are possibly the most attractive research on e-noses among the scientific community and have generated enormous interest worldwide [7,8,9]. Generally, this has been implemented by analyzing the response of the gas sensor array (providing multivariate signals) when exposed to chemical stimuli under well-controlled conditions (i.e., temperature, humidity, exposure time, etc.) [10]. Not surprisingly, many papers achieved more than 90% classification accuracy on various e-nose applications [11,12].

The concentration dependences of the most popular gas sensors (metal oxide semi-conductor, MOS) to be utilized in practical e-noses can be shown as a nonlinear power function form [13]. This means that the MOS sensors provide the intrinsic power law response of sensor electrical resistance to the concentrations of chemical stimuli. Moreover, when exposed to the mixture of several gases, the responses of the sensors become much more complicated. Therefore, designing and realizing a well-performed classification algorithm, which can simulate the relationship between the inputs (gas information) and outputs (electronic signals) of gas sensors well, is an effective approach to enhance the performance and enlarge the application field of the e-nose.

In this paper, we advocate the use of the well-known nonlinear approach, multiple kernel learning (MKL), to improve the performance of the extreme learning machine (ELM). Additionally, we introduce a simple but effective quantum-behaved particle swarm optimization (QPSO)-based weighed multiple kernel extreme learning machine (QWMK-ELM) model and apply it to e-nose data classification for the first time. This technique is attractive because it is simple and effective, furthermore, it is promising for future use in online applications. We investigate the influence of its main parameters due to the sensitivity of the algorithm to the parameter selection, i.e., the types and model parameters of the base kernels, the regularization parameter of the algorithm and the weighting coefficients used to construct the composite kernel. Furthermore, the performances of QWMK-ELM and other classification methods, namely, kernel ELM (KELM), ELM, support vector machine (SVM), k-nearest neighbors (KNN), probabilistic neural network (PNN), multi-layer perceptron with error back propagation algorithm (MLP-BP), and radial basis function neural network (RBFNN), are compared for both classification accuracy and execution time. Finally, we use one-way analysis of variance (ANOVA) to test whether the classification methods have a significant influence on classification accuracy rate.

The rest of the paper is organized as follows. Different classifiers of e-noses, especially the MKL-combined methods, are reviewed in Section 2. Section 3 introduces the methodology of the novel weighed multiple kernel extreme learning machine classification model based on QPSO. The two datasets used in our experimental validation are introduced in Section 4. Then, in Section 5, we present extensive experimentation results and necessary discussions. In Section 6, we draw the conclusion from the results and discussion in Section 5.

## 2. Related work

### 2.1. Classifiers

Scores of feasible and high-efficiency classification models have sprung up and been proven to be promising in e-nose applications over the past few decades [14,15,16,17,18]. They can be concisely categorized as two types [19,20]. One is the linear classifier. Early research by Martín et al. [21] utilized linear discriminant analysis (LDA) in an e-nose system to accomplish certain classification tasks about vegetable oils, which offers excellent classification and prediction competence. Song et al. [22] employed the partial least squares regression (PLSR) analysis to decide the predictive correlations between e-nose responses, the chemical parameters of the controlled oxidation of chicken fat, free fatty acid profiles, and gas chromatography-mass spectrometer (GC-MS) data and proved the promising application of e-nose systems in chicken fat oxidation control. Thaler et al. [23] used an e-nose with the logistic regression method to manage binary classification of bacteria data. Hassan et al. [24] combined a probabilistic framework with spike latency patterns in an e-nose for the quantification or classification of carcinogenic formaldehyde and used a naive Bayes classifier to evaluate the stochastic variability in the spike latency patterns. The linear classifier is relatively easy to establish and basically efficient, but functions in a limited manner when handling nonlinear problems.

As previous research work has demonstrated, the innate nonlinear attribute characterizes some e-nose data [6]. More specifically, when analyzing volatile organic compounds (VOCs), the data structure of the feature matrix derived from the e-nose response curves is nonlinear. Also, some exceptions will render the data structure nonlinear and complex [25]. To better cope with the nonlinear characteristic of the e-nose data, nonlinear classifiers are introduced into the e-nose applications. Artificial neural networks (ANNs), which typically possess nonlinear attributes, have been used in an e-nose system by Gardner et al. [26]. This work illustrated the superiority of the ANN over conventional methodologies. Pardo et al. [27] applied SVM to e-nose data classification and found this technique efficient, but strongly sensitive to the regularization parameter. Tang et al. [28] constructed an e-nose system with a KNN-embedded microprocessor for smell discrimination and demonstrated its excellent performance in distinguishing the chemical volatile of three kinds of fruits. In addition, the decision tree, which is a tree structure comprising internal and terminal nodes, was used in both the discrimination and dimensionality reduction of e-nose data by Cho et al. [29]. The nonlinear classifier can model the complicated nonlinear relationship between inputs and desired outputs and exhibits distinguished robustness and fault tolerance. Nevertheless, it shows delaying convergence and easily falls into local optima.

### 2.2. ELM

ELM, first put forward by Huang et al. [30] in 2004, is a single-hidden layer feedforward neural network (SLFN)-based learning algorithm, which selects hidden nodes randomly and computes the output weights of SLFNs analytically rather than tuning parameters iteratively. In this way, it exhibits excellent generalization performance at an exceedingly fast learning speed. Afterwards, Qiu et al. [31] applied ELM to e-nose data processing for both qualitative classification and quantitative regression of strawberry juice data and further concluded that ELM performed best in comparison to other pattern recognition approaches such as the learning vector quantization (LVQ) neural networks and SVMs. Over the last few decades, aware of the remarkable nature of ELM, a wide range of variants of ELM have been proposed to tackle the unconsidered or open questions remaining in this promising research field. As an example, fully-complex ELM (C-ELM) was designed to extend ELM from the real domain to the complex domain by Li et al. [32]. Similarly, Huang et al. [33,34] suggested incremental extreme learning machine (I-ELM), which incrementally increases randomly generated hidden nodes and the improved form of I-ELM with fully-complex hidden nodes to extend it from the real domain to the complex domain. They stated that I-ELM and C-ELM with fully-complex activation functions and with randomly-generated hidden nodes not relying on the training data can serve as universal approximators.

The kernel method, one of the various improvement methods for ELM, has aroused much interest and been utilized to promote a variety of systems ever since. Pioneering work by Huang et al. [35] succeeded in extending ELM to kernel learning, that is, ELM can use various feature mappings (hidden-layer output functions) involving not only random hidden nodes, but also kernels. In other words, in kernel ELM (KELM), which has been proven more efficient and stable than the original ELM, the hidden layer feature mapping is determined by virtue of a kernel matrix. Furthermore, KELM retains the characteristic of ELM, whose quantity of hidden nodes is randomly assigned. Then, Fernández-Delgado et al. [36] proposed a so-called direct kernel perceptron (DKP) on the basis of KELM. Fu et al. [37] achieved the fast determination of impact location using KELM. More recently, Peng et al. [38] perfectly applied KELM to the e-nose signals classification, which dramatically obtained high efficiency.

Despite the great applicability, however, a multitude of research works have demonstrated that the generalization ability of KELM is closely related to the kernel functions, and how to select or construct an effective kernel function that adapts to the practical problems is invariably a hot issue in the study of ELM. A simple KELM is generally implemented using a single kernel function, which can only reflect the characteristics of one class or one facet of data, and therefore is bound to trigger defects. The performances of KELMs with different kernels and model parameters are enormously different. The model parameters after training are still intensely sensitive to the samples. Consequently, the KELM has poor generalization ability and robustness due to the fixed form and a relatively narrow range of variation for a single kernel.

Recently, to better and more suitably address a specific problem, a more popular idea on kernel function establishment, called the multiple kernel learning (MKL) has been created and utilized. The MKL creates a feasible composite kernel by properly combining a series of kernels [39,40]. One of these kernels, the weighted kernel technique, has been further explored and has proved to be strikingly efficient in various studies. To name just a few, Sonnenburg et al. [41] offered an approach of convexly combining several kernels with a sparse weighting to overcome the problems within traditional kernel methods. Additionally, in 2014, Jia et al. [25] proposed a novel weighted approach to build the kernel function of kernel principal component analysis (KPCA) and utilized it in an e-nose to predict the wound infection ratings by extracting the data structure in the original feature matrix of wound infection data. They promoted the weighted KPCA (WKPCA) method and accomplished higher classification accuracy than that of many other classical feature extraction methods under the same conditions.

Moreover, research works have revealed the tremendous applicability of the weighted multiple kernel methodology in the field of ELM. Liu et al. [42] accomplished pioneering work and employed the weighted multiple kernel idea to solve two unconsidered issues in KELM and ELM: the ways of selecting an optimal kernel in a specific application context of KELM and coping with information fusion in ELM when there are various heterogeneous sources of data, and proposed sparse, non-sparse and radius-incorporated multiple kernel ELM (MK-ELM) methods. Furthermore, Zhu et al. [43] put forward the distance-based multiple kernel ELM (DBMK-ELM), which is a linear combination of base kernels and the combination coefficients are learned by virtue of solving a regression problem. It can attain an extremely fast learning speed and be adopted in both classification and regression, which was not accomplished by previous MK-ELM methods. Li et al. [44] proposed two formulations of multiple kernel learning for ELM by virtue of formulating it as convex programs, and thus, globally optimal solutions are guaranteed, which also proved to be competitive in contrast to the conventional ELM algorithm. In the learning of these different MK-ELMs, they are solved by constrained-optimization problems with different constraints. Usually, only the combination coefficients of base kernels and the structural parameters of classifiers (the output weights of SLFNs) are learned and analytically obtained by a matrix inverse operation and the regularization parameter *C* is specified arbitrarily [42,43]. In a different study, the regularization parameter *C* is jointly optimized with the combination coefficients of base kernels and the structural parameters of classifiers, which works better in most cases in comparison with the approach of pre-specifying *C* [44]. This means that all the algorithms regard the combination coefficients of base kernels (weights) as an inner parameter of SLFNs and obtain the optimal weights by serving them as constraints of the joint optimization objective function. In addition, all the algorithms do not optimize the kernel parameters of the base kernels, which are just specified as several special values. However, the kernel parameters of the base kernels have strong effects on the spatial distribution of the data in the high-dimensional feature space, which is defined by the kernel implicitly. On the other hand, the regularization parameter *C* is of great importance for the generalization performance of MK-ELMs. Consequently, the kernel parameters of the base kernels and the regularization parameter *C* need to be properly selected. All the MK-ELM algorithms emphasize the constrained-optimization problems for learning and lose sight of the effectiveness of intelligence optimization algorithms for parameter optimization. Furthermore, from a practical point of view, the application of MK-ELM in e-noses has not been explored.

## 3. Methodology

### 3.1. KELM

ELM [30,33,34,35,45] provided a generalized solution for SLFNs, whose hidden layer need not be tuned, and its learning speed is very fast. Compared with ELM, the KELM is able to ensure an implicit mapping using a kernel function exclusively instead of considering the mapping relationship definitely.

In general, suppose there are *N* arbitrary distinct samples $(x_{i},t_{i}),$ where $x_{i}={\left[x_{i1},x_{i2},\cdot \cdot \cdot ,x_{in}\right]}^{T}\in R^{n}$ is the *i*-th e-nose sample and $t_{i}={\left[t_{i1},t_{i2},\cdot \cdot \cdot ,t_{im}\right]}^{T}\in R^{m}$ is its corresponding sample class label. $t_{i}={\underset{k}{\left[0,\ldots ,0,1,0,\cdot \cdot \cdot ,0\right]}}^{T}\in {\left\{0,1\right\}}^{m}$ if $x_{i}$ belongs to the *k*-th (1≤k≤m) class. The number *n* denotes the dimensionality of the data $x_{i}$ and *m* denotes the dimensionality of its corresponding sample class label $t_{i}$, which is equal to the number of classes. Then, SLFNs and the activation function g(⋅)are modeled as:

(1) $$\begin{array}{l} \sum_{i=1}^{D}\beta_{i}g\left(w_{i}\cdot x_{j}+b_{i}\right)=o_{j},j=1,\cdot \cdot \cdot ,N\\ w_{i}={\left[w_{i1},w_{i2},\cdot \cdot \cdot ,w_{in}\right]}^{T},\beta_{i}={\left[\beta_{i1},\beta_{i2},\cdot \cdot \cdot ,\beta_{im}\right]}^{T} \end{array}$$

where $w_{i}$ is the weight vector connecting the *i*-th hidden neuron and the input neurons, $\beta_{i}$ is the weight vector connecting the *i*-th hidden neuron and the output neurons, D is the number of hidden neurons, $w_{i}\cdot x_{j}$ and $b_{i}$ denote the inner product of $w_{i}$ and $x_{j}$ and the threshold of the *i*-th hidden neuron, respectively. Finally, $o_{j}$ is the output vector of the *i*-th input sample.

Consequently, if an SLFN with *D* hidden nodes can approximate these *N* samples with zero error, which means that $\sum_{j=1}^{L}\left\|o_{j}-t_{j}\right\|=0$, there must exist $\beta_{i}$, $w_{i}$ and $b_{i}$ such that:

(2) $$\sum_{i=1}^{D}\beta_{i}g(w_{i}\cdot x_{j}+b_{i})=t_{j},j=1,\cdot \cdot \cdot ,N$$

which can be rewritten into a concise matrix form as:

(3) HB = T,

where:

(4) $$H\left(w_{1}\ldots w_{D},b_{1}\ldots b_{D},x_{1}\ldots x_{N}\right)=\left[\begin{array}{ccc} g\left(w_{1}\cdot x_{1}+b_{1}\right) & \ldots & g\left(w_{D}\cdot x_{1}+b_{D}\right)\\ \vdots & \ldots & \vdots \\ g\left(w_{1}\cdot x_{N}+b_{1}\right) & \ldots & g\left(w_{D}\cdot x_{N}+b_{D}\right) \end{array}\right]$$

(5) $$B=\left[\begin{array}{c} \beta_{1}^{T}\\ \vdots \\ \beta_{D}^{T} \end{array}\right]\;\mathrm{and}\;T=\left[\begin{array}{c} t_{1}^{T}\\ \vdots \\ t_{N}^{T} \end{array}\right]$$

Here, H is the hidden layer output matrix of the neural network. Then, we can use the Moore-Penrose generalized inverse of the hidden layer output matrix H labeled as $H^{+}$ to obtain a least-square solution as follows:

(6) $$B=H^{+}T$$

It is supposed to utilize a regularization coefficient C to calculate the output weights in terms of ridge regression theory:

(7) $$B=H^{T}{\left(\frac{I}{C}+{\mathrm{HH}}^{T}\right)}^{-1}T$$

where I represents an identity matrix. Based on Equation (7), the output function can be written as:

(8) $$f(x_{j})=h(x_{j})B=\left[\begin{array}{c} h(x_{j})h{\left(x_{1}\right)}^{T}\\ \vdots \\ h(x_{j})h{\left(x_{N}\right)}^{T} \end{array}\right]{\left(\frac{I}{C}+HH^{T}\right)}^{-1}T={\left(\begin{array}{c} k(x_{j},x_{1})\\ \vdots \\ k(x_{j},x_{N}) \end{array}\right)}^{T}{\left(\frac{I}{C}+K\right)}^{-1}T$$

where $h(x_{j})$ is the output of the hidden nodes by which the data from the input space is mapped into the hidden layer feature space. For arbitrary α-th and ρ-th input samples $x_{\alpha }(\alpha =1,2,\ldots ,N)$ and $x_{\rho }(\rho =1,2,\ldots ,N)$, a kernel function $k(x_{\alpha },x_{\rho })=h(x_{\alpha })h{\left(x_{\rho }\right)}^{T}$ can be used to define the mapping implicitly. Therefore, the index of the component of $f(x_{j})$ with the highest output value can be regarded as the predicted label of the sample $x_{j}$ [35].

### 3.2. Multiple Kernel Extreme Learning Machine

In the KELM, linearly inseparable patterns in the input space can be mapped into a high dimensional feature space and become linearly separable patterns using the nonlinear mapping of a kernel function, which can effectively achieve nonlinear classification. When applying the KELM, it is extremely crucial to choose the kernel $k(x_{\alpha },x_{\rho })$, which determines the model characteristics of the KELM in Equation (8) and the performance for classification tasks. The generalization ability of the KELM is closely related to kernel functions.

The KELM is implemented using a single kernel function, which can generally reflect the characteristics of partial data in the feature space. However, the performances of KELMs with different kernels and model parameters are of enormous difference, which determines the spatial distribution of the data in the high-dimensional feature space. The model parameters after training are still exceedingly sensitive to the samples. Consequently, the KELM has poor generalization ability and robustness due to the fixed form and a relatively narrow range of variation for a single kernel. For a non-flat distribution, finding suitable model parameters for KELM to fit both the rapid fluctuations and smooth changes well is an arduous task, since it is virtually impossible to describe a non-flat distribution well in any single feature space. However, taking multiple feature spaces into consideration may be a feasible solution, which is implicitly defined by virtue of a series of kernels with different parameters.

After the MKL was first proposed and of use in solving semi-definite programming (SDP) problems [39], researchers have more recently leveraged it to improve the performance of KELM to overcome the apparent deficiencies of KELM [35]. The MKL considers a group of mappings:

(9) $$\phi :\;x\in R^{n}\to \phi_{p}(x)\in F_{p}\;L\to F_{p}$$

In general, an optimal kernel is supposed to be any convex combination of a group of semi-definite functions, called base kernels. A weighted multiple kernels model can be defined as Equation (10), mapping the feature space into Hilbert spaces, leveraging the kernel trick [46]:

(10) $$\begin{array}{c} k\left(x_{i},x_{j};\lambda ,\Theta \right)=\sum_{q=1}^{Q}\lambda_{q}\langle \phi_{q}(x_{i}),\phi_{q}(x_{j}),\rangle =\sum_{q=1}^{Q}\lambda_{q}k_{q}(x_{i},x_{j};\theta_{q})\\ \sum_{q=1}^{Q}\lambda_{q}=1\;\mathrm{and}\;\lambda_{q}\geq 0\;\forall q \end{array}$$

where *Q* is a positive integer that is indicative of the number of base kernels, $k={\left\{k_{q}\right\}}_{q=1}^{Q}$ are previously defined base kernels, $\Phi ={\left\{\phi_{q}\right\}}_{q=1}^{Q}$ are the feature functions of the base kernels, $\Theta ={\left\{\theta_{q}\right\}}_{q=1}^{Q}$ are the set of kernel parameters, and $\lambda ={\left\{\lambda_{q}\right\}}_{q=1}^{Q}$ are the weighted coefficients of the base kernel combination. Equation (10) is equivalent to mapping the feature space into several subspaces, which are weighed consequently by the weights. Many characteristics of the optimal kernel are determined by the type of base kernel function. Mercer’s theorem [47] has already provided the characterization of a kernel function. Kernels can be divided into two categories: local kernels and global kernels [48]. For instance, the Gaussian kernel is a quintessential local kernel, in which only the data that are close to each other can influence the values of the kernel. Additionally, the polynomial kernel is a typical global kernel, which possesses an influence on the kernel values, allowing data points far away from each other. The composition of kernels may integrate the advantages of different kernels and has better performance than any single kernel.

Liu et al. [42] first proposed a multiple kernel extreme learning machine (MK-ELM) to address two issues in the research of ELM: (i) ELM pays little attention to optimizing the choice of kernels, and (ii) ELM lacks general a framework to integrate multiple heterogeneous data sources for classification. The approach regards the combination coefficients of base kernels (weights) as an inner parameter of SLFNs and obtains the optimal weights by serving them as a constraint of the optimization problem. The sparse MK-ELM, non-sparse MK-ELM, and radius-incorporated MK-ELM can be obtained from the uniform objective function form according to the different constraints as Equation (11):

(11) $$\begin{array}{l} \underset{\lambda }{\min}\underset{Β,\xi }{\min}\;\frac{1}{2}{\left\|B\right\|}_{F}^{2}+\frac{C}{2}\sum_{i=1}^{n}{\left\|\xi_{i}\right\|}^{2}\\ s.t.\;B^{T}g(x_{i};\lambda )=t_{i}-\xi_{i},\forall i,\;\sum_{q=1}^{m}\lambda_{q}=1,\;\lambda_{q}>0,\;\forall q \end{array}$$

where $g(x_{i};\lambda )(i=1,\ldots ,N)$ is the hidden layer output (feature mapping) corresponding to the training data $x_{i}$, B is the aforementioned output weights matrix of the SLFNs, ξ is the training error matrix on training data, $\xi_{i}={\left[\xi_{1i},\xi_{2i},\ldots ,\xi_{mi}\right]}^{T}(1\leq i\leq N)$ is the *i*-th column of ξ, and *C* is the regularization parameter which trades off the norm of output weights and training errors. $||\cdot |{|}_{F}^{2}$ is the Frobenius norm.

### 3.3. QPSO-Based Weighted Multiple Kernel Model

According to the above viewpoints, in this work, we empirically specified four different types of kernels (Gaussian kernel, polynomial kernel, sigmoid kernel, and wavelet kernel), which are applied as base kernels for multiple kernel combination and the model parameters Θ and λ, which need to be learned and optimized in order to realize an optimum mapping in the feature space. In our method, two base kernels that possess the same form, but different parameters are added in a weighted way as a new kernel function:

(12) $$\begin{array}{c} k_{\alpha \rho }(\Theta )=k(x_{\alpha },x_{\rho };\Theta )=\lambda_{1}k_{1}(x_{\alpha },x_{\rho };\theta_{1})+\lambda_{2}k_{2}(x_{\alpha },x_{\rho };\theta_{2})\\ \lambda_{1}+\lambda_{2}=1\;\mathrm{and}\;\lambda_{1}>0,\lambda_{2}>0 \end{array}$$

where the values of the weighting coefficients $\lambda_{1}$ and $\lambda_{2}$ are constant scalars, which are tuned in the training process and constitute a tradeoff of the two base kernels when mapping a given sample. The different values of $\lambda_{1}$ and $\lambda_{2}$ for different input space regions determine the characteristic of the weighted kernel. The weighting coefficient can be viewed as the rate of the relative contribution of one base kernel with respect to the other one. We employ various kernels with different parameters as base kernel functions to constitute a weighted multiple kernel and then implement the KELM shown in Equation (8).

As we all know, the performance of classifiers can be strongly affected by their parameters, which depend heavily on the training data. The kernel parameters of the base kernels have strong effects on the spatial distribution of the data in the high-dimensional feature space, which is defined by the kernel implicitly, and the regularization parameter *C* is of great importance for the generalization performance of MK-ELMs. Consequently, the kernel parameters and the regularization parameter *C* need to be properly selected. Besides, the weighting coefficients mentioned in Section 3.2 also need to be learned and optimized to indicate the importance of each kernel before the combination of the kernels and make the new combinatorial kernel obtain the best performance.

However, for the existing MK-ELM algorithms [42,43,44], the authors regard weights as an inner parameter of SLFNs and obtain the optimal weights by serving them as a constraint of the optimization problems. The kernel parameters of the base kernels and regularization parameter *C* are not optimized, they are just specified as several special values empirically. The algorithms emphasize on the constrained-optimization problems for learning but lose sight of the effectiveness of intelligence optimization algorithms for parameters optimization. Therefore, it is difficult to obtain the optimal model parameters and thus the best performance of the classifier.

In our method, the weights are not regarded as the inner parameters of SLFNs and the optimal weights are also not obtained by solving optimization problems. We regard the weights as an external parameter and optimize them by intelligence optimization algorithm, and then, the weighted sum of base kernels using the optimized weights is applied to construct the composite kernel function before implementing the KELM shown in Equation (8) with the composite kernel function. Meanwhile, the kernel parameters of each base kernel and the regularization parameter *C* are not specified arbitrarily, but optimized by an intelligence optimization algorithm simultaneously in order to obtain the optimal solution.

Quite a few intelligent optimization algorithms, including genetic algorithm (GA) [49,50], particle swarm optimization algorithm (PSO) [51,52], and quantum-behaved particle swarm optimization (QPSO) [53,54], etc., have been devoted to e-nose pattern recognition. In view of the complexity and especially the efficiency in our previous publication [38], QPSO [55,56] is leveraged to optimize the values of *C* in Equation (8), $\lambda_{1},\lambda_{2}$ in Equation (12), and the model parameters of the base kernels to constitute a weighted multiple kernel and then implement the KELM shown in Equation (8), which is named QPSO-based weighted multiple kernel extreme learning machine (QWMK-ELM).

QPSO integrates the quantum mechanics with the standard PSO by hypothesizing that each particle has a quantum state which can be represented by its wave function ψ(X,t) instead of the position and velocity in the standard PSO, where X=(x,y,z) is the position vector in three-dimensional space. The behavior of the quantum-behaved particle is different from the particle in standard PSO, where the position and velocity cannot be determined simultaneously. We can obtain the probability density of the appearance of the particle in a certain position according to ${\left|\psi (X,t)\right|}^{2}$, and thus obtain the probability distribution function. For the probability distribution function, through the Monte Carlo stochastic simulation method, the particle’s position is updated according to the following equation:

(13) $$x_{id}^{t+1}=p_{id}^{t}\pm \alpha \left|mbest_{id}^{t}-x_{id}^{t}\right|\times \ln(\frac{1}{u}),u=rand(0,1)$$

where $x_{i}^{t}={\left(x_{i1}^{t},\ldots ,x_{id}^{t},\ldots ,x_{iD}^{t}\right)}^{Τ}(i=1,2,\ldots M,1\leq d\leq D)$ means the position for the particle i at iteration t, where M is the number of the particles in the population and D is the dimension of the position (the number of the parameters that need to be optimized). α is the parameter of the QPSO algorithm and called the contraction-expansion coefficient, and $mbest_{id}^{t}$ is the average optimal position of all the particles and defined as $mbest_{i}^{t}=\frac{1}{M}\sum_{i=1}^{M}pbest_{i}^{t}$. $p_{id}^{t}$ is the local attractor and defined as $p_{id}^{t}=\varphi \times pbest_{id}^{t}+(1-\varphi )\times gbest_{gd}^{t},\varphi =rand(0,1)$. $pbest_{i}^{t}={\left(p_{i1}^{t},\ldots ,p_{id}^{t},\ldots ,p_{iD}^{t}\right)}^{T}$ is the local optimal position (the position giving the best fitness value) of particle i at iteration t and $gbest_{g}^{t}={\left(p_{g1}^{t},\ldots ,p_{gd}^{t},...p_{gD}^{t}\right)}^{T}$ is the global optimal position in the population at iteration *t*, where g is the index of the optimal particle among all the particles in the population. The overall optimization algorithm for solving the WMK-ELM incorporated QPSO is presented in Algorithm 1.
**Algorithm 1.** The QPSO-based WMK-ELM.1. **Initialize:** Swarm of QPSO: x={λ,C,Θ}, $pbest=pbest^{0}$, $gbest=pbest^{0}$ and t=0.2. **Construct:** Composite kernel: $K(\Theta )=\lambda_{1}K_{1}(\theta_{1})+\lambda_{2}K_{2}(\theta_{2})$.3. **Input**: K(Θ) and T.4. **Implement:** KELM by solving Equation (8).5. **Output:** Fitness value of QPSO (the classification accuracy).6. **Update:** Position: $\lambda^{t+1}$, $C^{t+1}$, $\Theta^{t+1}$ according to Equation (13),     Local and global optimum: $pbest^{t+1}$, $gbest^{t+1}$.     Iteration time: t=t+1.7. **Repeat:** Step 2 to step 8 until t=200.8. **End and Output:**
$gbest^{200}$ and the corresponding best fitness value.

## 4. Dataset

In this paper, two different datasets of gas sensor arrays are used, whose details have been elaborated in our previous publications [57,58], respectively. Hence, the materials and experiments are briefly revisited here to make the paper self-contained.

### 4.1. Dataset I

There are three indoor pollutant gases chosen as the targets, including toluene, formaldehyde, and carbon monoxide, which will be distinguished by the e-nose. The sensor array contains five sensors: three metal oxide semi-conductor (MOS) gas sensors (TGS 2201, TGS 2620, and TGS 2602) purchased from Figaro Company (Osaka, Japan), one temperature sensor, and one humidity sensor. The TGS 2201 has two outputs defined as TGS 2201A and TGS 2201B. The experimental platform is mainly made of an e-nose system, a personal computer, a temperature-humidity controlling system, a flow meter, and a vacuum pump. Before sampling experiments, the temperature and humidity of the chamber are set as 25 °C and 40%, respectively. Then, the experiment proceeds in terms of the following three procedures:

Procedure 1: Clean air circulates through all sensors for 2 min to acquire the baseline;

Procedure 2: Target gas is introduced into the chamber for 4 min;

Procedure 3: Clean air circulates through the array of the sensors for 9 min again to purge the sensors and allow them to recover to the baseline.

The specific distribution of the data is shown in Table 1.

### 4.2. Dataset II

The sensor array (sensing unit) is composed of eight MOS sensors with four different models and two different heater voltages, respectively. Two repetitions of the same sensor model are used in the array, and the two repetitions operate at two different voltages (5.00 V and 5.65 V, respectively) induced in the heater. Table 2 shows the details of the types and the heater voltages of sensors. Five independent detection units (e-nose systems) are used, following the same system design and implementation. Each unit is designed and built composed of eight MOS sensors (shown in Table 2) and is used for the detection of four different kinds of gases (ethylene, ethanol, carbon monoxide, and methane). The same experimental protocol is followed to measure the response of the five independent e-nose systems. Each day, one single unit is devoted to test the four types of gases with 10 different concentration levels and obtains 40 samples in total. Moreover, the five independent units are tested several times (a total of 16 days) over a 22-day period. Table 3 shows the day in which each unit is tested. The tests are performed on 16 of the 22 days and no tests are conducted on the 5th, 6th, 12th, 13th, 19th and 20th days. Overall, 640 samples are obtained.

The experiment proceeds according to the following three procedures:

Procedure 1: All sensors are exposed to clean air for 50 s to measure the baseline of the sensor response;

Procedure 2: The carrier gas is mixed with the selected volatile as the target gas and circulated during 100 s;

Procedure 3: The sensors are purged out by re-circulating only clean air during the subsequent 450 s.

The distribution of samples is shown in Table 4.

**Figure 1:**
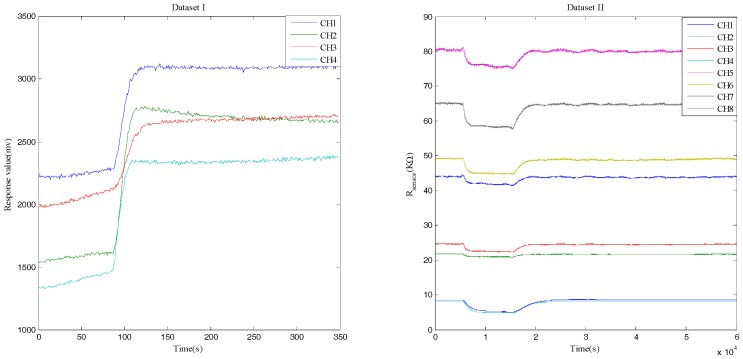
Response curves of the sensors in the two experiments. Dataset I has four channels (CH1–CH4), while Dataset II has eight channels (CH1–CH8).

Figure 1 manifests the representative response curves of the sensors for the two datasets. The responses of Datasets I and II are respectively voltage values and resistance values. Additionally, we take the steady-state values of responses for further research, or rather the peak values of responses in Dataset I and the valley values of responses in Dataset II.

## 5. Results and Discussion

Feature extraction methods have an important effect on the performance of the classifiers. They can be roughly divided into three categories: (1) extract piecemeal signal features from the original response curves of sensors, including steady-state response and transient responses such as peak values, integrals and derivatives etc.; (2) extract fitting parameters of a specific model which is used to fit the original sensor response curves; and (3) extract the transform coefficients of a specific transformation of the original sensor response curves such as the fast Fourier transform (FFT) and the discrete wavelet transform (DWT). Among the different features, the steady-state response (peak value) denotes the final steady-state feature of the entire dynamic response process in its final balance and reflects the maximum reaction degree change of sensors responding to odors. It is the most important information to distinguish different types and concentrations of gases and is usually used as the most common and simplest e-nose feature [59]. In this paper, we particularly emphasize the investigation of the capability of the proposed classification model, but not the comparison of the discrimination abilities of features. Therefore, the steady-state responses of the sensors are chosen as features for all the control methods in order to eliminate the effect of different features. Each of the same operations were carried out five times, and the average results of these are listed. The data has been divided into two subsets: the training set and the test set, as shown in Table 1 and Table 4. All procedures have been designed and tested with the same operation environment (MATLAB R2014a under the Windows Win10 (64-bit) operating system and 8 GB of RAM).

### 5.1. Performances of ELM with Different Models

**Figure 2:**
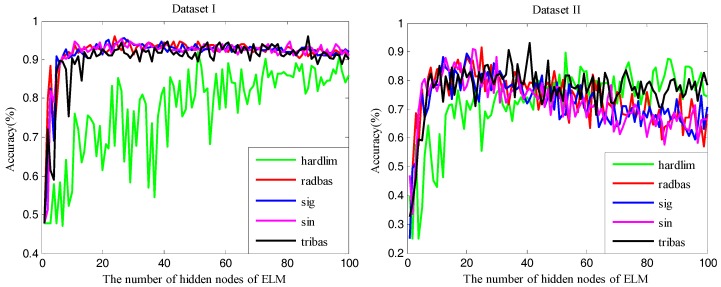
The classification accuracy of ELM in terms of 1–100 hidden nodes. The different colors of lines represent different activation functions. (Note: ELM represents the kernel extreme learning machine).

First of all, we studied the effect of different numbers of hidden nodes and types of activation functions on ELM. Figure 2 illustrates the performance of ELM with different numbers of hidden nodes for the two datasets. In Figure 2, it is obvious that the classification accuracies of ELM for both datasets are affected by not only the number of hidden nodes, but also the types of activation functions. For Dataset I, the classification accuracy of ELM increases quickly with the number of hidden nodes increasing from one to five, then goes up slightly until the number of hidden nodes reaches 25, and thereafter, it remains relatively steady, except for ELM using the hardlim activation function, which shows an overall rising trend. For Dataset II, the ELM using the hardlim activation function is also an exception and presents an overall rising trend. For ELMs with the other four kinds of activation functions, the classification accuracies climb up sharply until the number of hidden nodes reaches 10, obtain the highest accuracies with hidden nodes with the number ranging from 20–40, and then decline slowly. The generalization performance of ELM is affected greatly by the number of hidden nodes. It tends to become worse when too few or too many nodes are randomly generated. When the hidden nodes are too few it cannot learn the training data well. On the contrary, if the hidden nodes are too many, although the training error can be reduced, the training is easy to fall into the local minimum because of the too complicated model and the training accuracy is inconsistent with the test accuracy, i.e., overfitting. The number of hidden nodes is related not only to the number of nodes in the input/output layer, but also to the complexity of the problem to be solved and the type of the activation function, as well as the characteristics of the sample data. To avoid the overfitting phenomenon when training the model and to ensure a good generalization performance, we should make the structure of the model as compact as possible under the premise of meeting the accuracy requirement, that is, we should use as few hidden nodes as possible.

### 5.2. Performances of KELM and QWMK-ELM with Different Kernels

**Figure 3:**
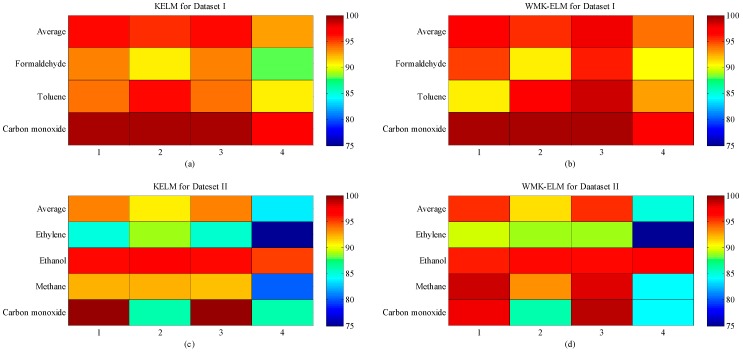
Classification results of QWMK-ELM and KELM for Dataset I and Dataset II. (Note: QWMK-ELM and KELM represent the quantum-behaved particle swarm optimization (QPSO)-based weighted multiple kernel extreme learning machine and kernel extreme learning machine respectively. In subplots (**a**) and (**c**), 1, 2, 3, and 4 represent Gaussian-kernel, polynomial-kernel, wavelet-kernel, and sigmoid-kernel, respectively. In subplots (**b**) and (**d**), 1, 2, 3, and 4 represent weighted-Gaussian-kernel, weighted-polynomial-kernel, weighted-wavelet-kernel, and weighted-sigmoid-kernel).

Before comparing the performance of different classifiers, we first compared the influence of four different kernel functions on QWMK-ELM and KELM for Dataset I and Dataset II (Figure 3). Comparing the results of the two datasets, some similarities in performance can be observed. The QWMK-ELM outperforms the KELM on the whole for both datasets no matter which type of kernel is leveraged. Besides, it is worth noting that the weighted wavelet kernel shows the best performance for both datasets. The sigmoid kernel presents the worst performance in KELM, and the weighted sigmoid kernel also has the worst result compared with the other three QWMK-ELM models.

### 5.3. Performances of Other Contrast Classification Models

**Figure 4:**
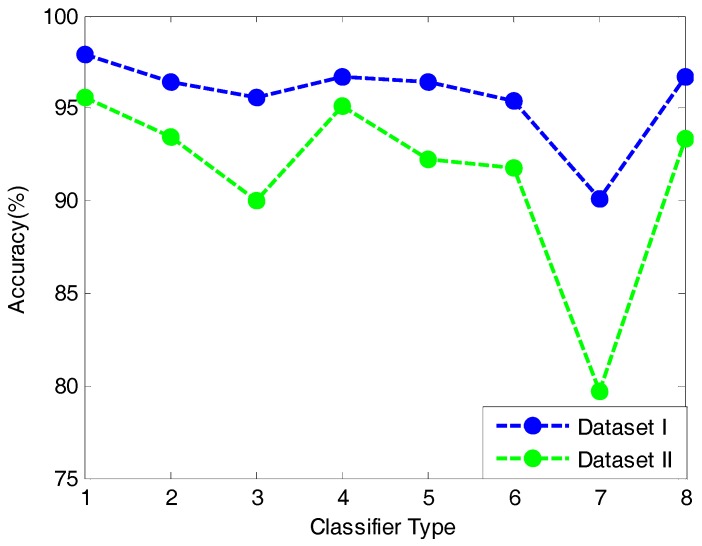
The overall trend of classification result among different methods of Dataset I and Dataset II. (Note: 1, QWMK-ELM; 2, KELM; 3, ELM; 4, MLP-BP; 5, RBFNN; 6, PNN; 7, KNN; 8, SVM).

In order to further certify the advantages of QWMK-ELM in classification, we perform an explicit comparison between QWMK-ELM and other classification methods. A host of different classifiers, i.e., ELM, MLP-BP, RBFNN, PNN, KNN, and SVM, are used as contrasts to demonstrate the validity of the proposed methods. Table 5 and Table 6 display the classification accuracies among different methods for Dataset I and Dataset II, respectively. From Table 5 and Table 6, we can see that all classifiers performed consistently well for both datasets, and most of them obtained accuracies of more than 90%, except for KNN for Dataset II, which has an accuracy lower than 80%. In addition, it is interesting to note that the overall trend of the classification results of different methods for Dataset I is in accord with that for Dataset II (shown in Figure 4). The proposed QWMK-ELM classifier consistently has the highest accuracies for both datasets and can attain 97.90% and 95.57% accuracies, respectively, while the KNN has the worst performances for both datasets, which are much lower than those of the other classification methods. This indicates that QWMK-ELM has an obvious advantage over other control classification methods. Comparing Table 5 and Table 6, we also can find that under the condition of uneven class sizes in the samples, the advantages of QWMK-ELM are more obvious, as it can choose a more appropriate kernel function to reflect the characteristics of the training data and thus has stronger generalization and robustness.

On the other hand, the execution time consumption of each classification method using the optimal model parameters obtained by QPSO is much different, which is shown in Table 7. MLP-BP, RBFNN, and PNN have much longer time consumptions than any other methods, which are from several times to more than 100-times those of the others. On the contrary, in sharp comparison to the better classification performance, the time consumption of QWMK-ELM is much less than the other classifiers, except for ELM, which means it has lower computational complexity. However, the increasing requirement for calculation compared with ELM represents a negligible loss as compared to the improvement of accuracy obtained. Besides, in order to show a notable improvement with respect the other methods clearly, we provide the relative improvement gain (RIG) of accuracy and execution time with respect the best competitors in Table 8 and Table 9. It is obvious that for both datasets, the MLP-BP is the best competitor with the highest accuracy among the several control methods. However, the execution times of the QWMK-ELM are over 150 times and 200 times better than the MLP-BP, and the RIGs in the classification accuracy are 1.23% and 0.43% for two datasets, respectively. This means that QWMK-ELM not only obtains higher accuracy but also has huge advantages in real-time application.

We use one-way analysis of variance (ANOVA) to test whether the classification methods have a significant influence on classification accuracy rate. Then, the test results from the two datasets can be obtained by statistical product and service solutions (SPSS), as shown as Table 10 and Table 11. It can be found that the values of *F* statistic are 1276.017 and 2042.881, respectively, which are significantly greater than 1 and the significance values are both 0.000. Give the level of significance α = 0.05, we can reject the null hypothesis and judge that there is a significant difference of accuracy rate under different classification methods.

**Figure 5:**
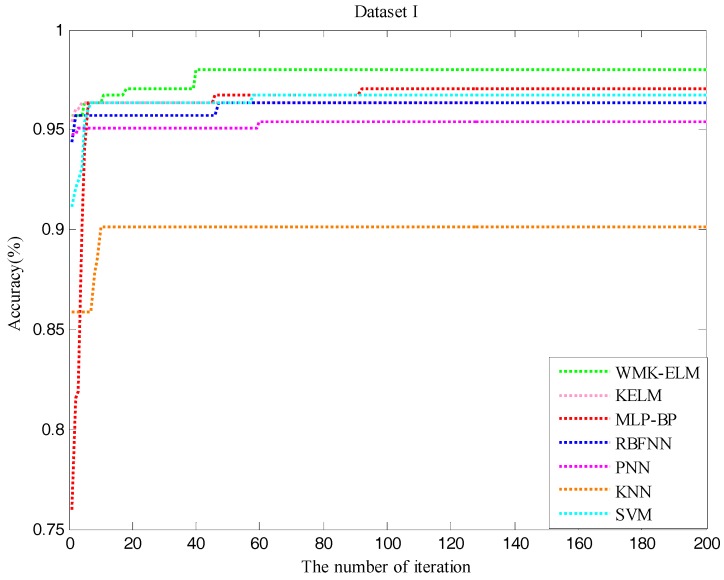
The accuracy of QWMK-ELM and the control classifiers in the process of optimization for Dataset I.

**Figure 6:**
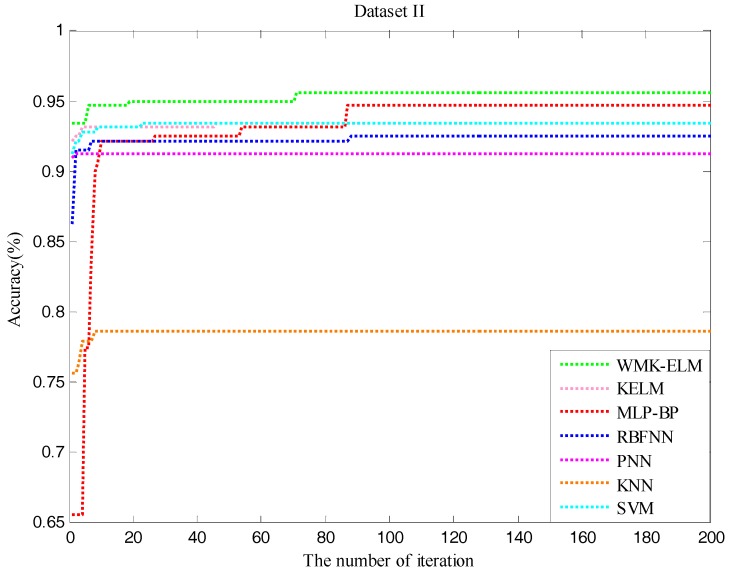
The accuracy of QWMK-ELM and the control classifiers in the process of optimization for Dataset II.

In order to visualize the process of the performance change, Figure 5 and Figure 6 illustrate the iterative process of both datasets when using QPSO to optimize the model parameters. It can clearly reflect how the classification rates change in the optimization procedure. According to the two figures, it seems to be able to draw conclusions that the control classification methods easily run into partial optimization at the early stage of the iteration, and the performance of all methods tends to be stable within 100 iterations. Although the times of total iterations are 200, it is not useful to enhance the classification effect with the increasing number of the iteration times, which only increases the time consumption of the parameter optimization.

## 6. Conclusions

In this paper, we explored a new framework to enhance the performance of ELM, which was combined with the weighted multiple kernels and the QPSO overmatching a generic single kernel. QWMK-ELM leveraged the weighted combination of multiple kernel functions and the QPSO for model parameters optimization. The weights were regarded as external parameters and optimized by QPSO, and then, the weighted sum of the base kernels using the obtained weights was applied as a kernel function. Meanwhile, the kernel parameters of each base kernel and the regularization parameter were not specified arbitrarily, but optimized by QPSO simultaneously. Therefore, it could better identify the characteristics of the data, increase the search space of the optimal kernel, enhance the robustness of the classifier, and thus further ameliorate the accuracy of classification. In order to further certify the efficiency of our method in classification, seven approaches, including ELM, KELM, MLP-BP, RBFNN, PNN, KNN, and SVM, were employed to deal with the same datasets and were compared with the QWMK-ELM. The results indicated the proposed model, QPSO-based WMK-ELM, outperformed KELM, ELM, BP, RBFNN, PNN, KNN, and SVM and had lower computational complexity. It was the first time that the multiple kernels ELM algorithm was applied to e-nose data, which shows promising performance. The results of the examination testified that the proposed QWMK-ELM offers a desired precision and efficiency in classification. It also had great potential to be optimized in a better way in future studies.

## Figures and Tables

**Table 1:** Amount of samples in Dataset I.

Gases	Training Set	Test Set
Carbon monoxide	145	145
Toluene	33	33
Formaldehyde	126	126
Total	304	304

**Table 2:** Models and Heater Voltages of MOS sensors.

Chanel	Sensor Type	Heater Voltage (V)
1	TGS2611	5.65
2	TGS2612	5.65
3	TGS2610	5.65
4	TGS2602	5.65
5	TGS2611	5.00
6	TGS2612	5.00
7	TGS2610	5.00
8	TGS2602	5.00

**Table 3:** Types and Heater Voltages of MOS sensors.

Number of Unit	Days Tested
Unit 1	4, 10, 15, 21
Unit 2	1, 7, 11, 16
Unit 3	2, 8, 14, 17
Unit 4	3, 9
Unit 5	18, 22

MOS: metal oxide semi-conductor.

**Table 4:** Amount of samples in Dataset II.

Gases	Training Set	Test Set
Ethylene	80	80
Ethanol	80	80
Carbon monoxide	80	80
Methane	80	80
Total	320	320

**Table 5:** Classification accuracy of QWMK-ELM and the control methods for Dataset I.

Class	Accuracy Rate (%)							
	QWMK-ELM	KELM	ELM	MLP-BP	RBFNN	PNN	KNN	SVM
1	99.31	99.31	98.48	96.55	98.62	**100.00**	95.86	98.62
2	98.79	93.94	92.73	**100.00**	96.79	93.94	96.97	93.94
3	**96.03**	93.65	93.02	93.65	93.65	90.48	91.75	95.24
Average	**97.90**	96.38	95.59	96.71	96.38	95.39	90.13	96.71

1, carbon monoxide; 2, toluene; 3, formaldehyde; MLP-BP, multi-layer perceptron with error back propagation algorithm; RBFNN, radical basis function neural network; PNN, probabilistic neural network; KNN, k-nearest neighbors; SVM, support vector machine.

**Table 6:** Classification accuracy of QWMK-ELM and the control methods for Dataset II.

Class	Accuracy Rate (%)							
	QWMK-ELM	KELM	ELM	MLP-BP	RBFNN	PNN	KNN	SVM
1	99.00	**100.00**	93.50	98.00	91.88	87.75	87.50	99.50
2	**98.25**	92.50	95.00	96.25	95.00	93.75	86.25	93.00
3	96.25	96.25	90.50	**97.50**	96.88	94.00	83.75	92.25
4	88.75	85.00	81.00	88.75	85.00	**89.50**	61.25	88.50
Average	**95.57**	93.44	90.00	95.16	92.20	91.78	79.69	93.32

1, carbon monoxide; 2, methane; 3, ethanol; 4, ethylene.

**Table 7:** Time consumption of QWMK-ELM and the control methods.

Classifier Type	Time Consumption (s)	
	Dataset I	Dataset II
QWMK-ELM	0.1221	0.0493
KELM	0.1024	0.0612
ELM	0.0394	0.0411
MLP-BP	18.6100	10.0449
RBFNN	3.2600	3.5389
PNN	2.3503	4.9807
KNN	0.5115	0.8992
SVM	0.2054	0.1060

**Table 8:** Accuracy and execution time improvement gain with respect to the best competitor for Dataset I.

Classification Method	Accuracy (%)	Execution Time (s)	Relative Improvement Gain	
			Accuracy	Execution Time
**QWMK-ELM**	97.90	0.1221	1.23%	151.42 times
**MLP-BP**	96.71	18.6100		

**Table 9:** Accuracy and execution time improvement gain with respect to the best competitor for Dataset II.

Classification method	Accuracy (%)	Execution Time (s)	Relative Improvement Gain	
			Accuracy	Execution Time
**QWMK-ELM**	95.57	0.0493	0.43%	202.75 times
**MLP-BP**	95.16	10.0449		

**Table 10:** ANOVA for Dataset I.

	Sum of Squares	df	Mean Square	F	Sig.
Between Groups	194.542	7	27.792	1276.017	0.000
Within Groups	0.697	32	0.022		
Total	195.239	39			

**Table 11:** ANOVA for Dataset II.

	Sum of Squares	df	Mean Square	F	Sig.
Between Groups	896.013	7	128.002	2042.881	0.000
Within Groups	2.005	32	0.063		
Total	898.018	39			
